# Supplementary material for: Pyruvate Dehydrogenase Kinase 1 inhibition mediated oxidative phosphorylation enhancement in cartilage promotes osteoarthritis progression
Source: BMC Musculoskelet Disord. 2023 Jul 20;24:597. doi: 10.1186/s12891-023-06585-6 (PMC10357736; doi:10.1186/s12891-023-06585-6)
Supplement: Supplementary file 1 — Additional file 1. [file 12891_2023_6585_MOESM1_ESM.zip › MF.Gsea.1653627316242/GOMF_OXIDOREDUCTASE_ACTIVITY_ACTING_ON_SINGLE_DONORS_WITH_INCORPORATION_OF_MOLECULAR_OXYGEN.html]

Details for gene set GOMF\_OXIDOREDUCTASE\_ACTIVITY\_ACTING\_ON\_SINGLE\_DONORS\_WITH\_INCORPORATION\_OF\_MOLECULAR\_OXYGEN[GSEA]

|  || Dataset | 55457\_collapsed\_to\_symbols.cls.cls #OA\_versus\_normal.cls.cls #OA\_versus\_normal\_repos |
| Phenotype | cls.cls#OA\_versus\_normal\_repos |
| Upregulated in class | OA |
| GeneSet | GOMF\_OXIDOREDUCTASE\_ACTIVITY\_ACTING\_ON\_SINGLE\_DONORS\_WITH\_INCORPORATION\_OF\_MOLECULAR\_OXYGEN |
| Enrichment Score (ES) | 0.34279487 |
| Normalized Enrichment Score (NES) | 1.1318536 |
| Nominal p-value | 0.29 |
| FDR q-value | 0.64603215 |
| FWER p-Value | 1.0 |
Table: GSEA Results Summary

  

Fig 1: Enrichment plot: GOMF\_OXIDOREDUCTASE\_ACTIVITY\_ACTING\_ON\_SINGLE\_DONORS\_WITH\_INCORPORATION\_OF\_MOLECULAR\_OXYGEN      
 Profile of the Running ES Score & Positions of GeneSet Members on the Rank Ordered List

  

| SYMBOL | TITLE | RANK IN GENE LIST | RANK METRIC SCORE | RUNNING ES | CORE ENRICHMENT || 1 | PTGS1 | prostaglandin-endoperoxide synthase 1 [Source:HGNC Symbol;Acc:HGNC:9604] | 163 | 0.845 | 0.0972 | Yes |
| 2 | RPE65 | retinoid isomerohydrolase RPE65 [Source:HGNC Symbol;Acc:HGNC:10294] | 308 | 0.743 | 0.1826 | Yes |
| 3 | ETHE1 | ETHE1 persulfide dioxygenase [Source:HGNC Symbol;Acc:HGNC:23287] | 572 | 0.632 | 0.2443 | Yes |
| 4 | ALOX5AP | arachidonate 5-lipoxygenase activating protein [Source:HGNC Symbol;Acc:HGNC:436] | 1665 | 0.404 | 0.2120 | Yes |
| 5 | HPD | 4-hydroxyphenylpyruvate dioxygenase [Source:HGNC Symbol;Acc:HGNC:5147] | 1681 | 0.402 | 0.2630 | Yes |
| 6 | TDO2 | "tryptophan 2,3-dioxygenase [Source:HGNC Symbol;Acc:HGNC:11708]" | 1783 | 0.387 | 0.3054 | Yes |
| 7 | ADI1 | acireductone dioxygenase 1 [Source:HGNC Symbol;Acc:HGNC:30576] | 2004 | 0.360 | 0.3351 | Yes |
| 8 | CDO1 | cysteine dioxygenase type 1 [Source:HGNC Symbol;Acc:HGNC:1795] | 2635 | 0.287 | 0.3235 | Yes |
| 9 | BCO1 | beta-carotene oxygenase 1 [Source:HGNC Symbol;Acc:HGNC:13815] | 2895 | 0.261 | 0.3373 | Yes |
| 10 | HAAO | "3-hydroxyanthranilate 3,4-dioxygenase [Source:HGNC Symbol;Acc:HGNC:4796]" | 3209 | 0.229 | 0.3428 | Yes |
| 11 | ALOX5 | arachidonate 5-lipoxygenase [Source:HGNC Symbol;Acc:HGNC:435] | 3776 | 0.179 | 0.3221 | No |
| 12 | ADO | 2-aminoethanethiol dioxygenase [Source:HGNC Symbol;Acc:HGNC:23506] | 5410 | 0.039 | 0.2004 | No |
| 13 | PIR | pirin [Source:HGNC Symbol;Acc:HGNC:30048] | 5743 | 0.012 | 0.1762 | No |
| 14 | ALOXE3 | arachidonate lipoxygenase 3 [Source:HGNC Symbol;Acc:HGNC:13743] | 7502 | -0.119 | 0.0552 | No |
| 15 | IDO1 | "indoleamine 2,3-dioxygenase 1 [Source:HGNC Symbol;Acc:HGNC:6059]" | 7782 | -0.140 | 0.0517 | No |
| 16 | ALOX12B | "arachidonate 12-lipoxygenase, 12R type [Source:HGNC Symbol;Acc:HGNC:430]" | 9617 | -0.284 | -0.0538 | No |
| 17 | HGD | "homogentisate 1,2-dioxygenase [Source:HGNC Symbol;Acc:HGNC:4892]" | 9669 | -0.289 | -0.0202 | No |
| 18 | ALOX12 | "arachidonate 12-lipoxygenase, 12S type [Source:HGNC Symbol;Acc:HGNC:429]" | 9683 | -0.290 | 0.0165 | No |
| 19 | ALOX15B | arachidonate 15-lipoxygenase type B [Source:HGNC Symbol;Acc:HGNC:434] | 11394 | -0.486 | -0.0531 | No |
| 20 | ALOX15 | arachidonate 15-lipoxygenase [Source:HGNC Symbol;Acc:HGNC:433] | 11405 | -0.488 | 0.0095 | No |
| 21 | PTGS2 | prostaglandin-endoperoxide synthase 2 [Source:HGNC Symbol;Acc:HGNC:9605] | 12641 | -0.820 | 0.0201 | No |
Table: GSEA details [plain text format]

  

Fig 2: GOMF\_OXIDOREDUCTASE\_ACTIVITY\_ACTING\_ON\_SINGLE\_DONORS\_WITH\_INCORPORATION\_OF\_MOLECULAR\_OXYGEN      
 Blue-Pink O' Gram in the Space of the Analyzed GeneSet

  

Fig 3: GOMF\_OXIDOREDUCTASE\_ACTIVITY\_ACTING\_ON\_SINGLE\_DONORS\_WITH\_INCORPORATION\_OF\_MOLECULAR\_OXYGEN: Random ES distribution      
 Gene set null distribution of ES for **GOMF\_OXIDOREDUCTASE\_ACTIVITY\_ACTING\_ON\_SINGLE\_DONORS\_WITH\_INCORPORATION\_OF\_MOLECULAR\_OXYGEN**
